# Understanding ecological systems using knowledge graphs: an application to highly pathogenic avian influenza

**DOI:** 10.1093/bioadv/vbaf016

**Published:** 2025-02-05

**Authors:** Hailey Robertson, Barbara A Han, Adrian A Castellanos, David Rosado, Guppy Stott, Ryan Zimmerman, John M Drake, Ellie Graeden

**Affiliations:** Department of Epidemiology of Microbial Diseases, Yale University School of Public Health, New Haven, CT 06510, United States; Center for Global Health Science and Security, Georgetown University Medical Center, Washington, DC 20007, United States; Cary Institute of Ecosystem Studies, Millbrook, NY 12545, United States; Cary Institute of Ecosystem Studies, Millbrook, NY 12545, United States; Center for Global Health Science and Security, Georgetown University Medical Center, Washington, DC 20007, United States; Institute of Bioinformatics, University of Georgia, Athens, GA 30602, United States; Center for Ecology of Infectious Diseases, University of Georgia, Athens, GA 30602, United States; Odum School of Ecology, University of Georgia, Athens, GA 30602, United States; Center for Global Health Science and Security, Georgetown University Medical Center, Washington, DC 20007, United States; Center for Ecology of Infectious Diseases, University of Georgia, Athens, GA 30602, United States; Odum School of Ecology, University of Georgia, Athens, GA 30602, United States; Center for Global Health Science and Security, Georgetown University Medical Center, Washington, DC 20007, United States; Massive Data Institute, Georgetown University, Washington, DC 20007, United States

## Abstract

**Motivation:**

Ecological systems are complex. Representing heterogeneous knowledge about ecological systems is a pervasive challenge because data are generated from many subdisciplines, exist in disparate sources, and only capture a subset of interactions underpinning system dynamics. Knowledge graphs (KGs) have been successfully applied to organize heterogeneous data and to predict new linkages in complex systems. Though not previously applied broadly in ecology, KGs have much to offer in an era when system dynamics are responding to rapid changes across multiple scales.

**Results:**

We developed a KG to demonstrate the method’s utility for ecological problems focused on highly pathogenic avian influenza (HPAI), a highly transmissible virus with a broad host range, wide geographic distribution, and rapid evolution with pandemic potential. We describe the development of a graph to include data related to HPAI including pathogen–host associations, species distributions, and population demographics, using a semantic ontology that defines relationships within and between datasets. We use the graph to perform a set of proof-of-concept analyses validating the method and identifying patterns of HPAI ecology. We underscore the generalizable value of KGs to ecology including ability to reveal previously known relationships and testable hypotheses in support of a deeper mechanistic understanding of ecological systems.

**Availability and implementation:**

The data and code are available under the MIT License on GitHub at https://github.com/cghss-data-lab/uga-pipp.

## 1 Introduction

The complexity of ecological systems is well-appreciated (Solé and Levin 2022). However, analysis in the field has largely relied on pairwise or targeted integration of specific subfields that hinges on previously established foundational knowledge about relationships between the topical domains (Silk *et al.* 2022). For example, the population dynamics of key species like pollinators and predators and the outbreak and pandemic spread of novel pathogens are examples of phenomena that emerge from the confluence of multiple simultaneous processes and non-linear relationships (Sánchez-Garduño *et al.* 2014, Brett *et al.* 2017, Tan *et al.* 2022). Within disease ecology specifically, there has been a move to include social science research, including policy analysis and survey data about human behavior such as vaccine hesitancy, with the more traditional quantitative and biological analyses performed by ecologists. To facilitate this interdisciplinary work into disease ecology, however, we need to operationalize new methods to integrate data from these systems and models across domains (Woldehanna and Zimicki 2015, Han and Drake 2016, Rivers *et al.* 2019, Todman *et al.* 2023, Friant 2024).

To successfully understand the relationships between these disparate systems, we need scalable methods to integrate broadly heterogeneous data and modeling methods. For example, remote sensing data from earth systems science can be combined with animal observation data to generate a composite understanding of phenology, abundance, migration, or extinction. Such synthetic analyses are increasingly common and offer a powerful deductive tool with which to better understand many dynamic processes in zoonotic disease outbreaks that are difficult to assess due in large part to data incompleteness.

Knowledge graphs (KGs) are graph-based structures that support data integration by encoding data points (nodes) and the relationships between them (edges), using semantic mappings to connect disparate data with shared underlying elements across domains and scales while avoiding taxonomic and unit misalignment. KGs are particularly effective in linking and structuring heterogeneous data to make them interoperable (i.e. connecting knowledge represented differently by different disciplines).

While not yet widely applied in ecology, the value of KGs has been demonstrated in various fields to explore interconnections between entities and detect anomalous patterns. For instance, biomedical KGs have been used for drug target discovery (Mohamed *et al.* 2019), prediction of potential drug–drug interactions (Ye *et al.* 2021), precision medicine (Chandak *et al.* 2023), and meta-analysis of diseases like COVID-19 (Pestryakova *et al.* 2022). Outside of biomedicine, KGs have integrated multi-source spatiotemporal data for natural disaster early warning systems and risk classification (Ge *et al.* 2022, Yang *et al.* 2022), humanitarian response efforts (Li *et al.* 2023), life sciences (Callahan *et al.* 2024), and environmental research (Zárate *et al.* 2019, Han *et al.* 2022). The expanding applications of KGs, coupled with recent advancements in machine-learning models, open up new potential for sophisticated analysis of heterogeneous datasets across disciplines and scales.

Here, we describe the application of KG methods to an ecological system and demonstrate the utility of the graph in ecology with a case study on “bird flu” (highly pathogenic avian influenza A virus or HPAI) to demonstrate how an ontology semantically connects data from heterogeneous sources to create a KG that can support statistical modeling and analysis of heterogeneous data about this system.

## 2 Methods

HPAI virus, specifically H5 and H7 subtypes, is an evolving threat to poultry, wildlife populations, and human health. In early 2020, an H5N1 lineage experienced a major resurgence and replaced the dominant H5N8 virus, spreading rapidly to all continents except for Australia (Xie *et al.* 2023). Since then, the H5N1 outbreak has caused unprecedented mass mortality in species that have never been detected with bird flu before—including many mammals (Graziosi *et al.* 2024). There is also evidence of significant mammal-to-mammal transmission, including among dairy cattle and detectable within milk, which has raised concerns about the rapid reassortment of the virus and its potential to cause a human pandemic (European Food Safety Authority (EFSA) *et al.* 2024, Petersen *et al.* 2024). Despite the virus’ zoonotic potential, relatively little is known about the dynamics of HPAI globally, such as how its viral evolution may impact geographic spread, host range, and the species-level attributes associated with infection (Runstadler and Puryear 2024). Therefore, we use the case of HPAI (Influenza A/H5N1) to demonstrate how KGs can support rapid outbreak analysis in a fragmented data environment, highlight knowledge gaps, and support hypothesis generation about cross-species transmission and future spread.

### 2.1 Building a KG for ecological analysis

**Figure 1.**
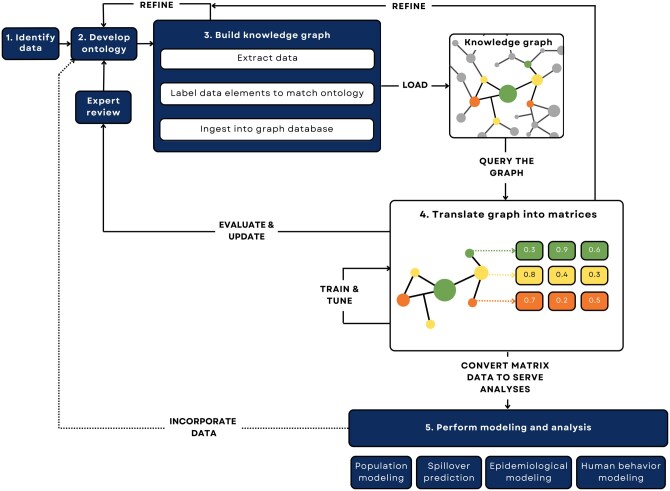
Process map depicting the steps to construct and use a custom knowledge graph from data identification to downstream machine-learning tasks. (1) Identify data requirements based on research questions, inventory data sources, and define shared data elements. (2) Develop an ontology by defining nodes as a combination of one or more shared data elements, defining edges as relationships between nodes, and using attributes to describe nodes or edges with additional detail. (3) Build the graph by extracting data from each source, labeling data elements from each source to match the ontology as a node, edge, or attribute, and ingesting data into the graph database. Note: this describes the extract–transform–load (ETL) pipeline. (4) Translate the graph into matrices for analyses, such as routine statistics or machine-learning models using knowledge graph embedding models or graph neural networks. (5) Perform analysis and modeling using the graph by converting the matrix data into parameters for population modeling, spillover prediction, epidemiological modeling, or human behavior modeling. Incorporate new, inferred knowledge into the graph if desired, refining the ontology and repeating the process as necessary.

Constructing and applying a KG to support scientific inquiry involves five steps (Fig. 1):

- Identify data sources to populate the graph.
- Develop an ontology that represents the real-world relationships that you are trying to describe and connects them with each of the chosen data sources.
- Extract, transform, and load data using graph database software to construct the KG.
- Embed the KG to test its coherence and train models on the structure and patterns of the graph.
- Perform downstream modeling and analysis.

#### 2.1.1 Identify data

To understand zoonotic disease system dynamics and model human spillover risk, we first defined key information requirements and compiled corresponding datasets with information about species traits, population demographics, pathogen characteristics, environmental factors, and human behavior into a data inventory. Of the data in the inventoried datasets, we identified six classes: reports, events, populations, taxa, geographies, and samples (Suchanek *et al.* 2019). All datasets contained at least one, and many had three or more of these classes represented, typically represented as columns or properties that were used as the basis for semantic classification.

Although 28 datasets were used to inform the shared data elements and ontology (see Supplementary Table S1), we prioritized seven key data sources to construct the initial graph given their relevance to zoonotic disease risk and shared concepts (e.g. classes) despite different taxonomies and structures. These were GeoNames (GeoNames n.d.), NCBI Taxonomy (Home—Taxonomy—NCBI n.d.), the Global Mammal-Parasite Database 2.0. (GMPD2) (Stephens *et al.* 2017), World Health Organization (WHO) FluNet (Flahault *et al.* 1998), World Organization for Animal Health (WOAH, formerly OIE) World Animal Health Information System (WAHIS) (World Animal Health Information System 2021), United Nations (UN) World Population Prospects (WPP) (World Population Prospects—Population Division—United Nations 2023), and the Coalesced Mammal Database of Intrinsic and Extrinsic Traits (COMBINE) (Soria *et al.* 2021).

GeoNames and NCBI Taxonomy were chosen for their stable unique identifiers (GeonamesId and TaxIds, respectively). These sources each have their own established ontologies and have been widely used to support KG and database development (Wardeh *et al.* 2015, Lin *et al.* 2021, Callahan *et al.* 2024).

GMPD2 provides data on the range of host–pathogen interactions for HPAI A(H5N1) and other pathogens (Stephens *et al.* 2017). While datasets like CLOVER (Gibb *et al.* 2021), EID2 (Wardeh *et al.* 2015), HP3 (Olival *et al.* 2017), and host–pathogen associations (Shaw *et al.* 2020) offer wider species coverage, GMPD2 uniquely provides publicly-available coordinates for each host–pathogen association. We prioritized a host–pathogen dataset with geographic coverage to counter the assumption that a host–pathogen association observed in one population applied universally to the species.

FluNet (Flahault *et al.* 1998) was used to obtain comprehensive weekly reports on human flu occurrences at the country level. WAHIS provides animal case data for 85 globally notifiable pathogens, including extensive reporting on HPAI (Terrestrial Code Online Access 2021, Lin *et al.* 2023). The data are updated in real time and accessible via a web portal (WAHIS n.d.). Use of data from the WAHIS platform requires the following statement: “WOAH bears no responsibility for the integrity or accuracy of the data contained herein, in particular due, but not limited to, any deletion, manipulation, or reformatting of data that may have occurred beyond its control.”

Human population and demographics were drawn from the UN WPP dataset (World Population Prospects—Population Division—United Nations 2023). The KG only contains human population data from UN WPP, capturing births, deaths, total population, net migration, and more. Currently, these data are only available at the country level.

Intrinsic and extrinsic trait data for species are provided by COMBINE (Soria *et al.* 2021). Measured properties include litter size, body mass, home range, diet, and more for analysis of species traits that may influence infectious disease risk (Han *et al.* 2020).

#### 2.1.2 Develop ontology

**Figure 2.**
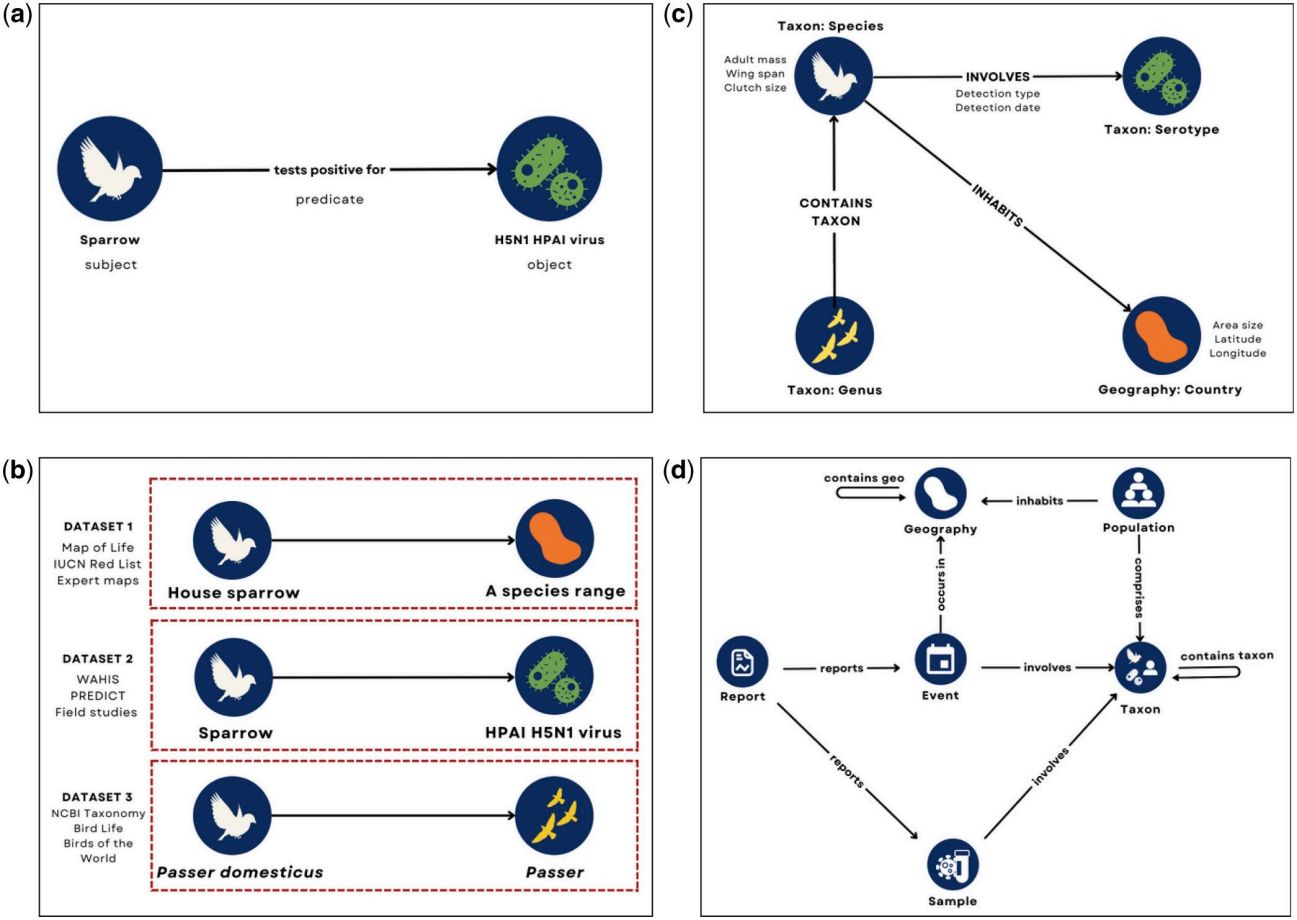
Visualizing a knowledge graph ontology. (a) Representing subject–predicate–object (SPO) triples in a graph format. (b) The current data ecosystem; each dotted box represents a type of tabular dataset that may contain many of the individual SPO triples. (c) A domain-specific ontology to represent an outbreak. Nodes (circles) represent instances of a class, with the bolded text above or below as labels with the node classes. To the left or right of each node are the properties of that node. (d) The full ontology and schema for the zoonotic risk knowledge graph. The graph schema adheres to the ontology.

A KG depends on its structure: a semantic ontology that describes the data that need to be linked. This ontology is an abstract representation of the real-world entities, attributes, and relationships in a given domain that provides a shared vocabulary to align and integrate data from a wide range of fields (e.g. ecology, epidemiology, genomics), even if those underlying data rely upon different taxonomies and formats (see Fig. 2). This domain-specific approach can offer significant value by capturing complex relationships, while also helping to build linkages between highly specialized fields.

For KGs, ontologies are built around three components: nodes, edges, and properties. Nodes represent an instance of a class (derived from data, sometimes called data elements), and edges are defined as the relationships between nodes. Attributes (or properties) capture additional values or details about the node or edge. These components are semantically combined to form “triples” which describe relationships between a subject (node), a predicate (edge), and an object (another node). For instance, the statement “A sparrow tests positive for H5N1 HPAI virus” is represented with sparrow as the subject node, H5N1 HPAI virus as the object node, and the relationship edge “tests positive for” as the predicate (Fig. 2a).

The current data ecosystem is characterized by both the lack of taxonomic interoperability across datasets despite shared elements (e.g. the use of “House sparrow”, “Sparrow” and “*Passer domesticus*” to represent the same species) and the lack of information about the relationships within the system (Fig. 2b). When data are structured as columns, each column represents a node in the current data ecosystem—but there are no “labeled” relationships. Rather, the user needs to intuitively know that the relationship between the “Host” column and the “Pathogen” column is that the host “carries” the pathogen (empirically) or the host was “detected with” the pathogen (in a specific sample).

With a KG ontology, relationships are semantically labeled to precisely define the connections, rather than needing to infer relationships across multiple disconnected datasets (also known as “rules mining” (Suchanek *et al.* 2019)). Any data referring to, e.g. a species—regardless of the naming convention—would map to the same node (Fig. 2c). This enables KGs to achieve interoperability and interpretability across fields without mandating the use of specific terms or measurement types in the source data. Thus, KGs with robust ontologies are a foundation for growth as new data becomes incrementally available.

While network models have a similar structure with multiple nodes and edges, all of the edges represent one kind of relationship (e.g. network models of host–pathogen associations (Dallas *et al.* 2019)). KGs, on the other hand, support an effectively infinite set of relationships to capture and analyse nuanced processes—though in practice, this scalability is bounded by computational and data constraints (Fig. 2d).

Given the nuance of ecological relationships, including the influence of higher-order interactions, domain experts curated the taxonomy and ontology to ensure a comprehensive and accurate representation of ecological interactions and phenomena. Drawing from the six node classifications defined in step one, each was defined as an entity with nine relationship types (edges) describing the zoonotic risk domain, including ecology, demographics, outbreak events, and more (Fig. 2d). The node classifications are defined in Table 1.

**Table 1.** Node classifications.

Node type	Definition
Event	Any ecological phenomena that occur at a time in a place, such as an outbreak
Report	Documentation of the event, structured or unstructured
Taxon	Any rank of a group of organisms (not individuals) from the NCBI Taxonomy
Geography	Any coordinate location or place name/type, e.g. continent, landmark, ecological niche
Population	A subset of individuals of a species that occupy and interact within an area
Sample	Any biological specimen

‘Events’ are any ecological phenomena that occur at a time in a place, such as an outbreak. ‘Reports’ are documentation of that event, structured or unstructured. Most report nodes are connected to an event; however, museum collections, archives, and passive surveillance efforts may exist only as reports with no event. For instance, in this graph, FluNet and WAHIS populate both reports and events, whereas GMPD2 populates just reports because the dataset describes known host–pathogen associations not specific to a discrete event.

‘Taxon’ nodes represent a rank of a group of organisms (not individuals) from the NCBI Taxonomy and are used to reconcile schematic and semantic differences arising from ecological and health-related data sources. The name of the species mentioned in a report is mapped to the TaxId with nodes for host and pathogen taxa added only when explicitly defined in a report and when host or pathogen names have a match in NCBI. We err on the side of inclusion, using the most specific name available (e.g. preserving finer classification below species level). Names that cannot be reconciled with the NCBI Taxonomy were matched to the next highest taxonomic rank (e.g. the next least specific term) such that a report identifying Influenza A (H5) with no neuraminidase subtype is matched to Influenza A. Because some species might be either host or pathogen given some specific context, taxa were not defined by this characteristic.

Reports and events are each defined by place and time. ‘Geography’ nodes can be as large as a continent or ecological niche and as small as an individual coordinate. These nodes are hierarchical and contain all smaller areas within them, including different types of boundaries (e.g. countries may contain states, counties, species ranges, and more). GeoNames was used to match places in the data to nodes in the KG based on name or reverse geocoded points.

Events affect ‘populations’, which live in primary geographies and other locations where they spend time whether to overwinter or through which they migrate. Population-specific traits are stored as metadata associated with corresponding taxon nodes.

‘Sample’ nodes represent data about biological specimens and laboratory analysis on those specimens and may include one or more taxa, particularly if capturing both host and pathogen species from a specific infection.

#### 2.1.3 Build the KG

The process of building a KG based on the ecology-specific ontology described above is most simply described as a version of an ETL pipeline: extraction (E), transformation (T), and loading (L). ETL pipelines are the standard data engineering process of identifying, curating, and integrating datasets; KGs extend this method to facilitate interoperability between heterogeneous datasets.

In the context of KG development, extraction refers to the accessing of datasets for integration into the KG (e.g. API, remote or local .csv files). Once retrieved, we performed data cleaning which included removing incomplete rows, dropping columns not relevant to the KG, resetting data types, and splitting strings.

In this case, transformation refers to the alignment of case data to other ecological, geographic, or socioeconomic data. To support analysis across domains, host and pathogen names were matched to their taxonomic species in NCBI, and the data collection locations were standardized (e.g. using Geonames). For the former, we used NCBI e-fetch to identify host and parasite names either using taxonomic ID, if available, or fuzzy matching, where not. Each taxon was defined as a node with attributes including the taxon’s scientific name, other name synonyms, taxonomic ID, and rank. Geographic coordinates, placenames, or polygon shapefiles were then geocoded or reverse geocoded by match to their GeonamesId. This same reconciliation process for all names (e.g. host, pathogen) and places (e.g. coordinates, country names) was completed for each of the six datasets included in the KG. All other data elements were labeled to match the ontology as a node, edge, or attribute. Any unlabeled data were excluded from the KG. See Supplementary Table S2 for a list of all nodes, edges, and their properties.

Data were loaded into Neo4j graph data software using Python (3.9) and Neo4j (5.10). A series of queries were written in Cypher to reference the labels set for each data element and create the triples defined in the ontology. When run, these queries match and merge existing node patterns, and set new attributes and labels (see Supplementary File S2 for the specific queries). The data can be loaded in full at each run, or a singular source can be updated to reduce the number of times that the KG must be reconstructed. See the GitHub repository code for more detailed information (https://github.com/cghss-data-lab/uga-pipp).

#### 2.1.4 Embedding: translate the graph into matrices

KG embeddings are a type of machine-learning model that supports the extraction and quantification of semantic information. These embedding models traverse structural patterns in the graph to learn continuous vector representations (embeddings) of nodes and edges, which are then translated into numerical representations (matrices) (Suchanek *et al.* 2019, Mohamed *et al.* 2021). Embedding models must be iteratively trained on the data, though subsets may be used to allow for incremental training with quicker and more efficient adjustment of parameters (Lerer *et al.* 2019, Suchanek *et al.* 2019). Embeddings are useful to validate semantic coherence, and necessary for more complex or probabilistic reasoning tasks, such as link prediction, because they can identify and quantify the strength of relationships based on explicit and implicit patterns in the graph, without depending entirely on deterministic rules defined in the ontology (such as with semantic reasoning technologies). In ecology, e.g., KG embeddings can integrate phylogenetic, abundance, and functional trait data to capture the effects of evolutionary or trait-matching processes on network structure (Strydom *et al*. 2021, 2023). By embedding such metadata into a continuous vector space, these models allow for hypothesis testing, such as determining whether phylogenetic or functional signals drive ecological interactions, or predicting unseen relationships within ecological networks (Poisot *et al.* 2023, Strydom *et al.* 2023).

Because KGs are relationship-centric, each type of embedding model is tailored to a particular type or direction of the relationship between nodes. We chose four embedding models each suited to different types of relationships to test which would perform best on our graph: TransE, ComplEx, DistMul, and RotatE (Wang *et al.* 2017). We chose TransE (Bordes *et al.* 2013) because it is well-suited for one-to-one relationships using simple vectors, ComplEx (Trouillon *et al.* 2016) because it uses complex vectors (with real and imaginary numbers) to capture multi-dimensional relationships, DistMul (Yang *et al.* 2017) because it uses a bilinear diagonal model which is effective for one-to-many relationships, and RotatE (Sun *et al.* 2019) because it is adept at handling many-to-many relationships using rotational transformations.

The KG embeddings were trained and optimized with the PyTorch library (PyTorch n.d.) using a subset of the data, encompassing Geography, Taxon, and Report nodes populated from GMPD2 and FluNet. We then compared the KG embeddings against the standard metric of hits@*k*, which measures how often the models ranked the correct relationships (true triplets) within the top *k* highest-scoring (most likely) triplets (Dai *et al.* 2020). A higher hits@*k* score indicates better model proficiency in predicting and differentiating the connections between various entities within the KG (Onuki *et al.* 2017, Yin *et al.* 2018, Dai *et al.* 2020). In the biomedical domain, precision often remains below 0.2, as observed in Hetionet and BioKG, making values above this range desirable (Himmelstein *et al.* 2017, Lerer *et al.* 2019, Rivas-Barragan *et al.* 2022).

**Figure 3.**
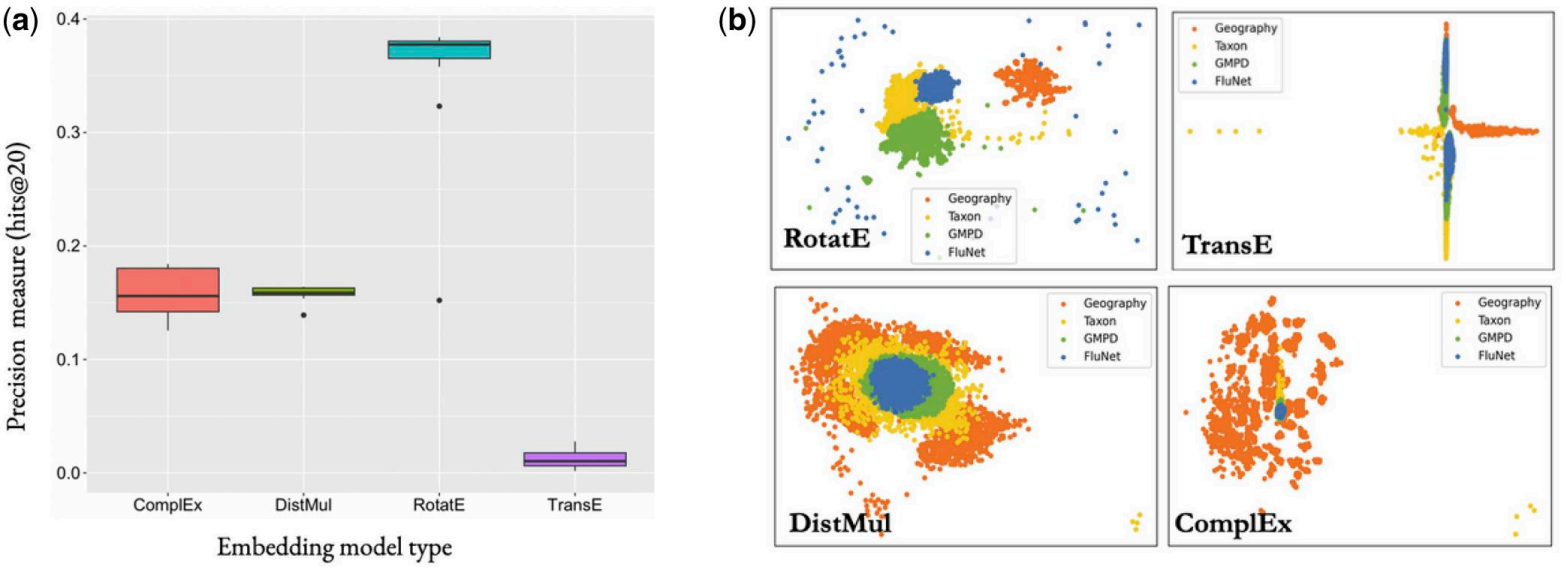
Comparison of four different KG embedding models’ performance in their ability to distinguish between node types based on connectivity patterns within the graph using data from GeoNames, NCBI Taxonomy, GMPD, and FluNet. (a) Precision of KG embedding models (ComplEx, DistMul, RotatE, TransE), measured by hits@20. The *y*-axis indicates their precision, measured by hits@20. RotatE performed the best, and TransE performed the worst. (b) KG embeddings projected in 2D space, with embedding-predicted similar nodes and relationships clustered together and dissimilar ones further apart in space.

The results, as depicted in Fig. 3a, show the precision of each model in learning the relationship types within the ontology for this graph. RotatE performed at ∼0.4 precision, indicating that it is most able to accurately predict triples, likely due to the number of many-to-many relationships within the KG (Fig. 3b). The strong performance of RotatE suggests that the ontology is semantically coherent because the structure of the graph was successfully represented with high precision, meaning it had a sufficient level of structural consistency and logical validity to be represented accurately in a continuous vector space and have good predictive performance. This suggests the resulting model can be used to provide reliable probabilistic insights for downstream machine-learning tasks. By comparison, TransE performed the worst with less than 0.05 precision, likely because there are no one-to-one relationships represented in the ontology for it to learn (Fig. 3b). DistMul and ComplEx performed similarly; both cluster similar nodes together with accuracy, but they are unable to differentiate nodes by type (Fig. 3b).

## 3 Results

### 3.1 Applications of the KG to HPAI

The current outbreak of HPAI A/H5N1 has pandemic potential having caused unprecedented mass mortality events in wild bird populations, evolved mammal-to-mammal transmission occurring between numerous species, and human cases observed on dairy farms (Klaassen and Wille 2023, Leguia *et al.* 2023, Garg *et al.* 2024, Sah *et al.* 2024). One of the most important areas for future research on the reassorted HPAI A/H5 lineage involves understanding if, when, and where transmission may spread to mammal populations (Nishiura *et al.* 2023). Identifying mammals most heavily affected in recent and historical influenza outbreaks, and evaluating geographic patterns and characteristics of those outbreaks, may indicate relationships that can help disentangle cross-species transmission pathways and future species of concern for spillover. While there have been studies of specific populations, there has not been a global assessment of where H5 cases are occurring (Nishiura *et al.* 2023) in part because the data are difficult to integrate from existing sources (Klaassen and Wille 2023) and it is traditionally not feasible to analyse patterns across numerous outbreaks given the size of the data.

**Figure 4.**
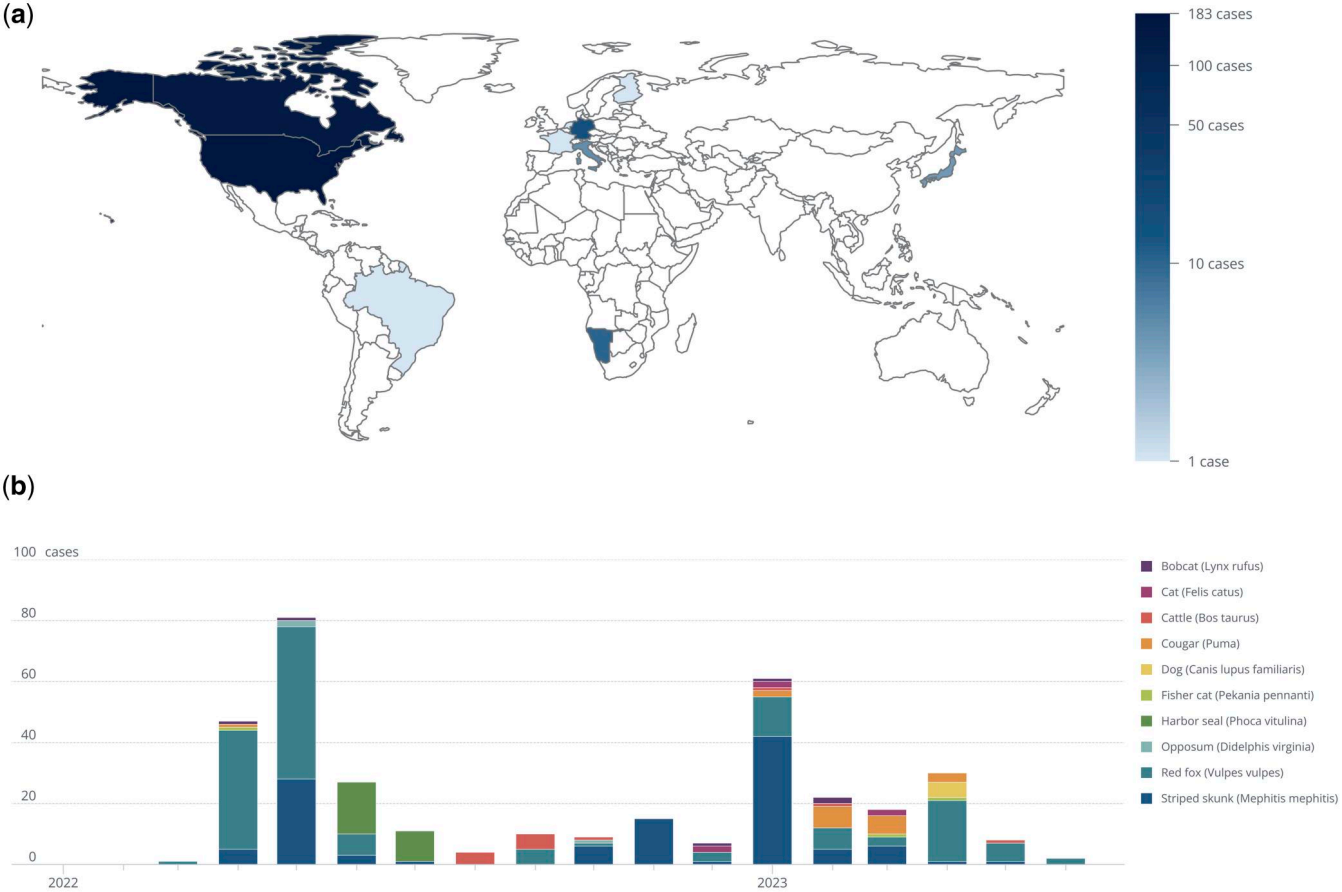
KG merges data about HPAI A/H5N1 in mammals across geographies, time, taxonomies, and scales using relationship-centric queries. (a) Map of total HPAI A/H5N1 cases in mammals as reported in WAHIS from 1 January 2020 to 1 July 2023, excluding cases in humans. (b) Monthly counts of HPAI A/H5N1 cases from January 2020 to July 2023 for the top 10 mammal species by number of cases over time.

We use the KG to integrate avian and mammalian case data associated with the HPAI A/H5N1 lineage for the ongoing outbreak and identify shared locations between highly affected mammals to better understand which places (and which other species within those places) may be at risk of spillover. To achieve this, we wrote a single query that links HPAI A/H5N1 cases to pathogen and host species information through events and geographic locations, allowing identification of shared locations between highly affected mammal species. It filters cases by date and location to determine regions with the highest numbers of overlapping cases, which can indicate potential spillover risks to other species within those areas (see Supplementary File S3). Using this query, we find that from January 2020 to July 2023, H5N1 cases in mammals were reported in nine countries, with over 90% of cases reported in the United States and Canada (Fig. 4a). The outbreak spans 21 mammal species, with cases initially reported in *Vulpes vulpes* (Red fox) by Japan and Denmark to WAHIS on 31 March 2022 and 1 April 2022, respectively (Fig. 4b). Additional H5N1 cases were reported among captive and wild red fox in the United States and Canada in the following weeks. In early April, cases were reported in *Mephitis mephitis* (Striped skunk) exclusively in the United States and Canada (Fig. 4b). These two species encompass over 70% of total H5N1 cases reported to WAHIS in mammals, which suggests that these species are key carriers of the virus in affected regions. However, this pattern may also reflect case ascertainment bias, as surveillance efforts or detection methods might disproportionately focus on these species, potentially underrepresenting other affected mammals. By identifying these species and their case locations, the query results elucidate new hypotheses to explore where and how these species might interact with domesticated species or humans, thus informing risk assessments and public health strategies.

**Figure 5.**
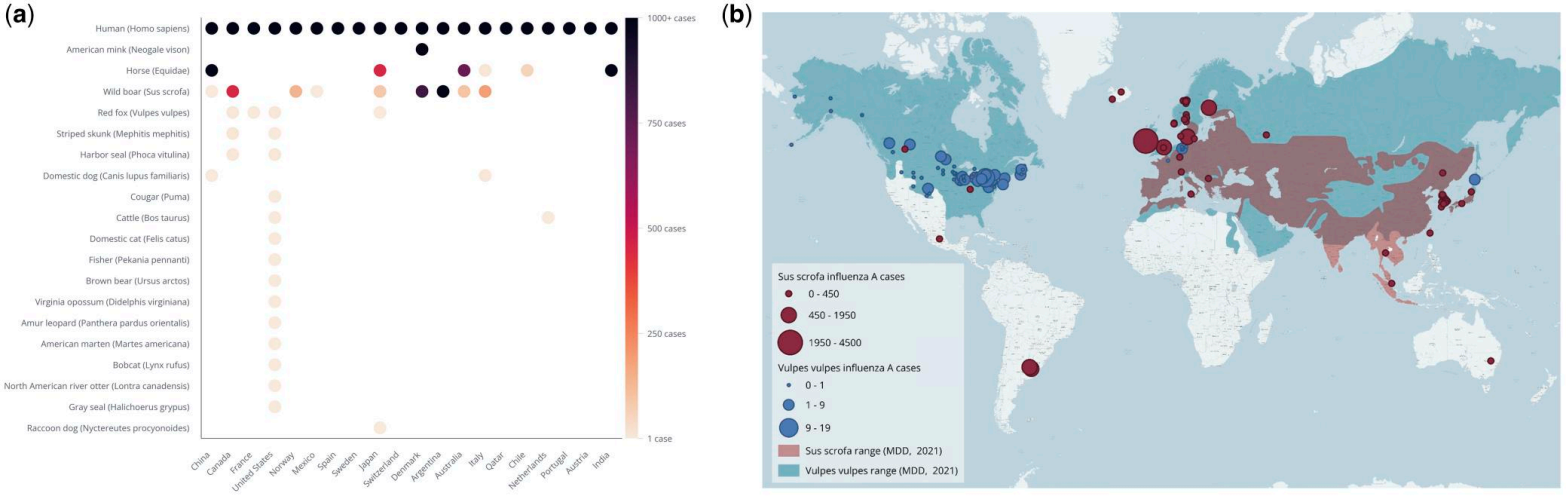
Integrating data for rapid exploratory analysis of HPAI A/H5N1. (a) Heatmap showing total flu cases for all mammal species from 2005 to 2023, for the top 20 countries by total human cases over that time. (b) Geographic distribution of cases against species range map for *Vulpes vulpes* (red fox) and *Sus scrofa* (wild boar), due to their identification as the top two non-domesticated species in (a).

To expand the analysis across human and animal networks to identify places of risk and gaps in surveillance, we analysed FluNet and WAHIS data together to explore historical influenza outbreaks involving mammals and identify locations where cross-species transmission, particularly relevant to humans, may occur based on first-order interactions (Fig. 5a). Subsequently, we identified specific geographical locations that may warrant additional data collection (Fig. 5b).

Among the 20 countries with the highest burden of human influenza, *Sus scrofa* (wild boar) and red fox were the top two mammalian species affected by influenza A (Fig. 5a). Influenza A cases in boars and red foxeswere reported in Canada, the United States, and Japan. Notably, boar was not a species reported with HPAI A/H5N1, whereas red foxhad the highest number of reported cases. Analysing the geographical range of these host species revealed significant overlap in western Europe which may be ripe for additional surveillance, especially given the proximity of these animals to humans (Fig. 5b). Countries in this region also have a high burden of human influenza. Simultaneous infections in human and animal populations that interact frequently can intensify selection pressure on the virus, creating opportunities for potential reassortment events at the animal–human interface (Li *et al.* 2010, Octaviani *et al.* 2010, Reperant *et al.* 2014).

Once familiar with the query syntax (e.g. Cypher), querying the KG requires only knowledge of the relationships or entities of interest; repeated queries allow for rapid evaluation of data across multiple datasets at different scales. These findings allow for exploratory analysis of HPAI A/H5N1 prevalence by host. Species distribution, ecological niches, and trophic level data (e.g. from COMBINE, the IUCN Red List, Map of Life, and food web models) could be used to identify potentially susceptible or at-risk hosts. Integrating genomic data could improve the biological accuracy of host–pathogen interactions and outbreak models.

## 4 Discussion

Decision-makers face the urgent need to identify when and where new cross-species transmission events are likely to occur with the ongoing outbreak of HPAI A/H5N1 and take immediate action to get ahead of a possible public health emergency. Challenges with gaining these insights in the status quo are that researchers are limited to asking pairwise questions (e.g. which host carries a particular pathogen?) and that the right questions must be identified and defined before analysis. A KG, on the other hand, allows researchers to answer questions they may not even consider asking of the data, propose new hypotheses, and evaluate new areas of research. We demonstrate how researchers can query to look for patterns across historical outbreaks in terms of the host species infected, populations affected, and regions impacted to determine possible characteristics of future outbreaks of that (or similar) pathogens. The KG helps query and visualize these connections, as well as taxonomic lineages and geographic trees, to support data exploration to suggest other relationships not captured directly in the data through inference and reasoning. This approach is useful in data-limited settings or for undersampled pathogens or host species.

The KG method offers these new insights more rapidly and in a more scalable manner than traditional analysis techniques (e.g. relational or tabular formats). By collating the data in the KG, we were able to analyse species distribution, geospatial coverage, and temporal distribution from a single source, at a more granular level than available via the WAHIS data download alone and without needing to reconcile to new scales or taxonomies. For instance, we were able to align host and causative agent names in WAHIS (often reported as common names) to the NCBI Taxonomic backbone to facilitate linkages to other data sources and previous outbreaks involving these species. Affected places, reported by coordinate points, were rolled up to the country level by reverse geocoding and navigating through administrative levels in GeoNames.

This flexibility is in contrast to a relational or tabular format, which enforces a rigid structure that requires researchers to specify the exact columns and filtering criteria. Any data that does not fit this predefined structure is not surfaced, obscuring potentially valuable information—especially if the researcher does not know to ask for it. So, while it is possible in a traditional format to produce the types of analysis performed in Figs 4 and 5, scaling up the research questions necessitates either restructuring the query over and over, re-analysing the dataset, and seeking new data sources. These tasks are often time-consuming and labor-intensive, and may not yield comprehensive insights.

In a tabular data approach, producing the analysis shown in Fig. 5 would likely involve at least eight distinct, manual steps often requiring joins and manual reconciliation (see Table 2). Such a process requires intermediate results and data wrangling to combine insights, which not only leads to slower workflows but also more potential for error and difficulties with reproducibility. Meanwhile, the graph-based approach streamlines the process significantly with a single, reusable query by matching data to predefined patterns and variables from the ontology. Though the query can be long and appear complex if specific filters are desired (e.g. returning cases from countries where a specific virus infected a mammal between certain dates, as in Supplementary File S3), a researcher who is familiar with their underlying ontology will be able to traverse seamlessly between interconnected data like ecological or epidemiological networks, returning the required data in the desired format without multiple intermediate steps. For instance, the graph handles intersections between geopolitical and natural boundaries, maintaining the integrity of point data throughout the transformation. Taxonomic integration is native to the graph, allowing for a smooth transition between common and scientific names.

**Table 2.** Steps for tabular versus graph-based data approach.

Tabular approach	Graph-based approach
1. Create mappings between taxonomies in different data sources, including for species and places	Mappings completed on data ingest with ontology
2. Reverse geocode coordinates from WAHIS for each influenza outbreak in mammal species, and aggregate up to the country-level	
3. Sum all human cases of influenza (e.g. Influenza A, Influenza B, H5N1, etc.) by country from 2005 to 2023	All matching data retrieved with a single query with no transformation required
4. Sum all mammal cases of influenza by mammal species and by country from 2005 to 2023	
5. Order countries by the number of human cases and select the top 20	
6. For each country, return the total number of mammal cases by species using the species’ common name	
7. For specific species (wild boar and red fox), re-retrieve and merge outbreak point data	

**Figure 6.**
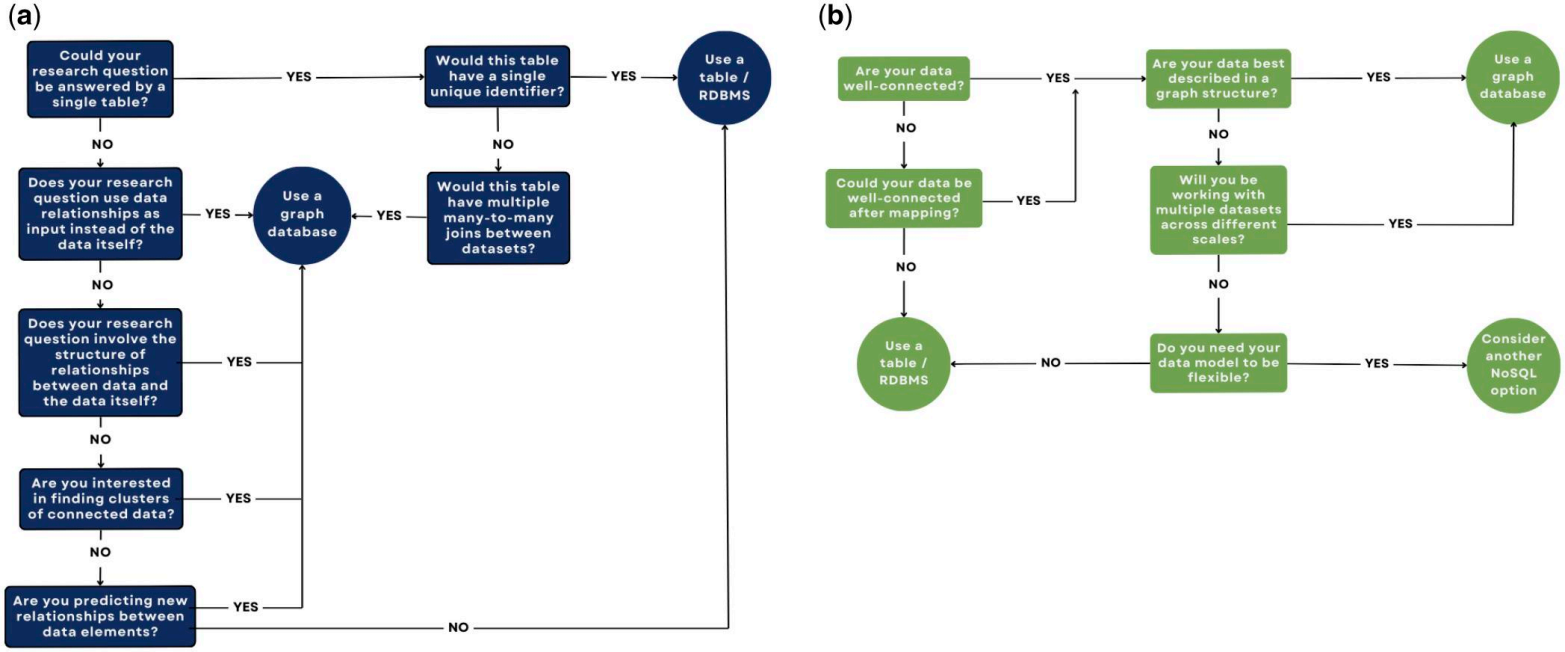
Decision tree for choosing a relational versus a graph database. (a) Decision tree based on the type of research question being asked. (b) Decision tree based on the structure of the data being used.

Broadly, relational database management systems (RDBMS) perform best in queries requiring row-by-row access; graph databases, in contrast, perform best in queries focusing on the structure of relationships between data (Codd 1970). As a result of these two distinct paradigms of data storage, there are differences in the types of research questions that perform best in each database type. RDBMS is the best choice when there are few many-to-many joins between datasets, there is a clear index or collection of tables that could answer the question, or the data structure itself is not of interest. While graph databases can handle many of the same questions that RDBMS can, they tend to be more difficult to work with (requiring specialized query languages) and perform slower in group by, aggregation, and sort operations. However, graph databases exhibit better performance and scalability in traversal queries, multi-table joins, and pattern matching (Patras *et al.* 2021). Decision trees for when to use an RDBMS or a graph database based on the research questions and structure of the data are shown in Fig. 6.

**Figure 7.**
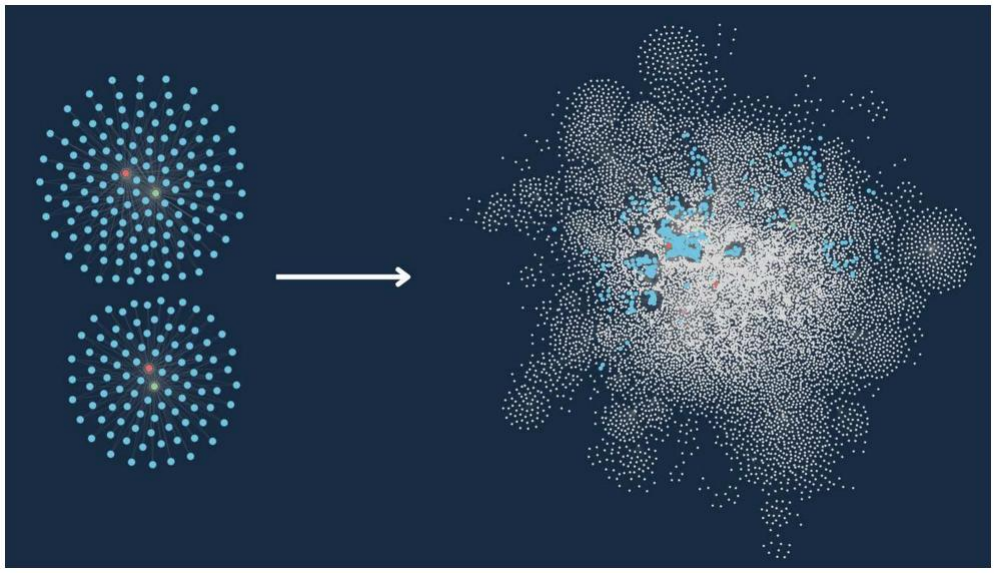
Using path expansion within the graph to surface linked data. Expanding from influenza outbreaks involving *Sus scrofa* (wild boar) and *Vulpes vulpes* (red fox) within the graph.

Beyond the ability to rapidly execute more complex queries for targeted results (represented by the colorful nodes in Fig. 7), the KG ontology provides a cloud of interconnected data that enables deeper exploration and insight suggested by data integration (represented by the white nodes in Fig. 7). This is in contrast to traditional methods of network science in ecology, such as bipartite networks, which typically focus on pairwise relationships (e.g. host–pathogen interactions) and are less capable of traversing cross-disciplinary boundaries.

In the KG approach, querying a single host species surfaces not just its direct associations—such as compatible pathogens or prior outbreaks—but its broader taxonomic lineage, phenotypic traits, diet, habitat preference, and geographic range. These interconnected layers enable researchers to uncover patterns and hidden relationships across scales, such as aligning local biosecurity measures (e.g. at the admin 1 level) with host–pathogen data reported at varying scales, from county or country-levels to species’ geographic ranges that span borders.

Furthermore, the KG approach enables scalable hypothesis generation and data integration across disciplinary boundaries even when sampling is sparse—a broadly recognized challenge in ecological methods. This flexibility proves particularly valuable in interdisciplinary response contexts where timely evidence generation and decision-making is crucial. For example, with HPAI A/H5N1, pinpointing the next possible mammalian host remains challenging given how unpredictable the virus’ evolution has been (Xie *et al.* 2023). However, by looking for highly central host species from past outbreaks of HPAI A/H5/H7 and other influenza A virus subtypes, we can begin to understand which host–pathogen associations are over-represented and those that are conspicuously absent in the data. This helps to prioritize species for testing given the limited surveillance resources, and adapt strategies for evolving ecological and epidemiological conditions.

There are, however, several challenges to working with KGs beyond the need for technical expertise in constructing them. For instance, domains with sparse data may not be suitable for downstream modeling. When domains are inadequately represented in the KG, advanced analytical techniques have reduced pattern recognition, leading to incomplete or biased insights. Therefore, relationships inferred from sparse data should be approached with skepticism and validated by subject-matter experts before being accepted as new “knowledge” to incorporate back into the graph. Furthermore, decision-makers should be careful to generalize beyond the resolution of the data provided in the graph, as conclusions drawn from aggregated or low-resolution data may not accurately reflect local dynamics or variations that could significantly impact the outcome of specific interventions or policies. Instead, they should use the available data to guide resource allocation and prioritize regions or populations where more granular data can be collected.

## 5 Conclusion

The results presented here are based on query-based approaches, where existing, verified relationships within the KG were extracted to identify ecological and epidemiological patterns with regard to HPAI. These query-based methods operate within the bounds of structured, curated data and do not involve machine-inferred relationships. However, as KG applications continue to evolve, embedding models (as those discussed in Section 4) present an opportunity for future researchers to infer new relationships based on the structural patterns of the graph, thus facilitating hypothesis generation in data-limited areas. In this context, explainable AI techniques could be useful in validating and interpreting these inferred relationships, such as to assess which parameters most informed the newly predicted relationship or outcome (Kelly *et al.* 2023). This application would help to validate insights before they are incorporated as new knowledge.

The broader implications of employing KGs and ontologies for ecology—especially when combined with embedding-based inference artificial intelligence—enable researchers to break out of traditional data silos and readily integrate expertise and data from between subfields and disciplines. For instance, integrating pathogen genomics, transmission vectors, and environmental condition data could improve synthesis and prediction for many emerging infectious diseases beyond influenza. KGs could also be used, e.g. to integrate climate models, land use data, species occurrence records, and ecological niche assessments to predict future impacts on biodiversity given climate change driven shifts in species distributions. Furthermore, if additional datasets on host-pathogen interactions at different spatial resolutions (e.g. from CLOVER, HP3, and Shaw *et al.* 2020) were incorporated into the KG, researchers could more precisely quantify the variability in surveillance coverage across geographic scales. This added context would enable domain experts to investigate how data gaps might bias the outcomes of predictive models or limit our understanding of pathogen transmission dynamics.

As the world continues to face rapid global changes that grow more interconnected, the ability to integrate and analyse multiscale data and models will be crucial in predicting and mitigating complex systemic impacts (Pokutnaya *et al.* 2023). KGs hold promise in addressing this challenge.

## Supplementary Material

vbaf016_Supplementary_Data

## Data Availability

No original data were generated during the course of this study, but all data and code required to reproduce the knowledge graph and figures here are available on GitHub at https://github.com/cghss-data-lab/uga-pipp.
