# Supplementary material for: Understanding ecological systems using knowledge graphs: an application to highly pathogenic avian influenza
Source: Bioinform Adv. 2025 Feb 5;5(1):vbaf016. doi: 10.1093/bioadv/vbaf016 (PMC11879169; doi:10.1093/bioadv/vbaf016)
Supplement: vbaf016_Supplementary_Data [file vbaf016_supplementary_data.docx]

**Supporting information**

**Supplementary Table 1: Data inventory**

| Data source name | URL |
| --- | --- |
| Area of Habitat maps for terrestrial birds and mammals | https://www.nature.com/articles/s41597-022-01838-w |
| ASM Mammal Diversity Database (MDD) | https://www.mammaldiversity.org/index.html |
| AVONET | https://onlinelibrary.wiley.com/doi/full/10.1111/ele.13898 |
| BACI-CEPII database | http://www.cepii.fr/CEPII/en/bdd_modele/bdd_modele_item.asp?id=37 |
| BV-BRC | https://www.bv-brc.org |
| Carnivore Pathogen- and Parasite-Species Pairings | https://www.sciencedirect.com/science/article/pii/S147149222100204X#s0055 |
| COMBINE: A Coalesced Mammal Database of Intrinsic and Extrinsic Traits | https://esajournals.onlinelibrary.wiley.com/doi/full/10.1002/ecy.3344 |
| EltonTraits | https://esajournals.onlinelibrary.wiley.com/doi/10.1890/13-1917.1 |
| FAOSTAT Crops and Livestock Data | https://www.fao.org/faostat/en/ |
| GenBank | https://www.ncbi.nlm.nih.gov/genbank/ |
| GeoNames (Polygons) | https://www.geonames.org/export/ws-overview.html |
| GISAID EpiFlu: Global Initiative on Sharing Avian Influenza Data | https://www.gisaid.org/ |
| Global Infectious Diseases and Epidemiology Network (GIDEON) | https://www.gideononline.com/ |
| Global Mammal Parasite Database (GMPD) | https://esajournals.onlinelibrary.wiley.com/doi/full/10.1002/ecy.1799 |
| Global patterns and predictors of avian population density | https://doi.org/10.1111/geb.13688 |
| Human Footprint Index | https://www.nature.com/articles/s41597-022-01284-8 |
| IUCN Red List | https://www.iucnredlist.org/ |
| NCBI Taxonomy | https://ftp.ncbi.nih.gov/pub/taxonomy/ |
| SafeGraph Core Places Dataset | https://docs.safegraph.com/docs/places |
| Unacast: Location Data | https://www.unacast.com/ |
| United Nations (UN) World Population Projections | https://population.un.org/wpp/ |
| USDA Animal and Plant Health Inspection Service (APHIS) Confirmations of Highly Pathogenic Avian Influenza in Commercial and Backyard Flocks | https://www.aphis.usda.gov/aphis/ourfocus/animalhealth/animal-disease-information/avian/avian-influenza/hpai-2022/2022-hpai-commercial-backyard-flocks |
| USDA Animal and Plant Health Inspection Service (APHIS) Confirmations of Highly Pathogenic Avian Influenza in Wild Birds | https://www.aphis.usda.gov/aphis/maps/animal-health/wild-bird-avian-flu-surveillance |
| USGS Wildlife Health Information Sharing Partnership Event Reporting System (WHISPers) | https://whispers.usgs.gov/home |
| VIRION | https://viralemergence.github.io/virion/ |
| WAHIS: World Animal Health Information System | https://wahis.woah.org/#/events |
| WHO FluNet | https://www.who.int/tools/flunet |
| Zoonomia Project | https://zoonomiaproject.org/the-data/ |

**Supplementary Table 2: Nodes, edges, and properties**

| Label | Properties | Property data source(s) |
| --- | --- | --- |
| Event (node) | Data source; Description; Start date; End date; Samples collected; Samples processed; Event ID | FluNet; WAHIS |
| Geography (node) | Data source; Place name; Feature class name; Feature code; Feature code name; Geoname ID; Latitude; Longitude | GeoNames |
| Population (node) | Data source; Report ID; Total population; Median age; Natural change in population; Population change; Births; Deaths; Life expectancy; Infant deaths; Under five deaths; Net migration | UN World Pop |
| Report (node) | Data source; Reason for report; Report date; Report description; Report ID | FluNet; WAHIS |
| Sample (node) | Data source; Sample ID; NCBI Accession ID; Collection date; Collection institution | GMPD-2 |
| Taxon (node) | Data source; Name; Rank; Tax ID; Adult mass; Brain mass; Adult body length; Adult forearm length; Maximum longevity; Days to maturity; Female days to maturity; Male days to maturity; Days to first reproductive cycle; Gestation length; Teat number; Litter size; Litters per year; Interbirth interval; Neonate mass; Weaning age; Weaning mass; Generation length; Dispersal; Density; Home range; Social group size; Percent of diet from invertebrates; Percent of diet from vertebrates; Percent of diet from plants; Percent of diet from mammals and birds; Percent of diet from reptiles and amphibians; Percent of diet from fish; Percent of diet from unknown vertebrates; Percent of diet from dead organisms; Percent of diet from fruits; Percent of diet from nectar and pollen; Percent of diet from seeds; Percent of diet from other plant materials; Number of dietary categories in diet; Upper elevation boundary; Lower elevation boundary; Altitude breadth; Habitat breadth; Hibernation; Freshwater habitat; Marine habitat; Terrestrial non-volant habitat; Terrestrial volant habitat; Island dwelling; Dissected by mountains; Glaciation; Fossoriality source; Trophic level; Foraging stratum; Activity cycle; Island endemicity | NCBI Taxonomy; COMBINE |
| CONTAINS_GEO (edge) | N/A |  |
| CONTAINS_TAX (edge) | N/A |  |
| INVOLVES (edge) | Observation date; Observation type; Positive cases; Samples collected; Samples processed; Subtype; Deaths; Role; Wild species | GMPD-2; FluNet; WAHIS |
| OCCURS_IN (edge) | N/A |  |
| REPORTS (edge) | N/A |  |

**Supplementary File 1: Embeddings**

**Supplementary File 2: Queries for ingest**

*COMBINE:*

UNWIND $Mapping as mapping

MERGE (tax:Taxon {name : mapping.iucn2020_binomial})

ON MATCH SET

tax.adult_mass_g = toFloat(mapping.adult_mass_g),

tax.brain_mass_g = toFloat(mapping.brain_mass_g),

tax.adult_body_length_mm = toFloat(mapping.adult_body_length_mm),

tax.adult_forearm_length_mm = toFloat(mapping.adult_forearm_length_mm),

tax.max_longevity_d = toFloat(mapping.max_longevity_d),

tax.maturity_d = toFloat(mapping.maturity_d),

tax.female_maturity_d = toFloat(mapping.female_maturity_d),

tax.male_maturity_d = toFloat(mapping.male_maturity_d),

tax.age_first_reproduction_d = toFloat(mapping.age_first_reproduction_d),

tax.gestation_length_d = toFloat(mapping.gestation_length_d),

tax.teat_number_n = toFloat(mapping.teat_number_n),

tax.litter_size_n = toFloat(mapping.litter_size_n),

tax.litters_per_year_n = toFloat(mapping.litters_per_year_n),

tax.interbirth_interval_d = toFloat(mapping.interbirth_interval_d),

tax.neonate_mass_g = toFloat(mapping.neonate_mass_g),

tax.weaning_age_d = toFloat(mapping.weaning_age_d),

tax.weaning_mass_g = toFloat(mapping.weaning_mass_g),

tax.generation_length_d = toFloat(mapping.generation_length_d),

tax.dispersal_km = toFloat(mapping.dispersal_km),

tax.density_n_km2 = toFloat(mapping.density_n_km2),

tax.home_range_km2 = toFloat(mapping.home_range_km2),

tax.social_group_n = toFloat(mapping.social_group_n),

tax.dphy_invertebrate = toFloat(mapping.dphy_invertebrate),

tax.dphy_vertebrate = toFloat(mapping.dphy_vertebrate),

tax.dphy_plant = toFloat(mapping.dphy_plant),

tax.det_inv = toFloat(mapping.det_inv),

tax.det_vend = toFloat(mapping.det_vend),

tax.det_vect = toFloat(mapping.det_vect),

tax.det_vfish = toFloat(mapping.det_vfish),

tax.det_vunk = toFloat(mapping.det_vunk),

tax.det_scav = toFloat(mapping.det_scav),

tax.det_fruit = toFloat(mapping.det_fruit),

tax.det_nect = toFloat(mapping.det_nect),

tax.det_seed = toFloat(mapping.det_seed),

tax.det_plantother = toFloat(mapping.det_plantother),

tax.det_diet_breadth_n = toFloat(mapping.det_diet_breadth_n),

tax.upper_elevation_m = toFloat(mapping.upper_elevation_m),

tax.lower_elevation_m = toFloat(mapping.lower_elevation_m),

tax.altitude_breadth_m = toFloat(mapping.altitude_breadth_m),

tax.habitat_breadth_n = toFloat(mapping.habitat_breadth_n),

tax.hibernation_torpor = mapping.hibernation_torpor,

tax.freshwater = mapping.freshwater,

tax.marine = mapping.marine,

tax.terrestrial_non_volant = mapping.terrestrial_non_volant,

tax.terrestrial_volant = mapping.terrestrial_volant,

tax.island_dwelling = mapping.island_dwelling,

tax.disected_by_mountains = mapping.disected_by_mountains,

tax.glaciation = mapping.glaciation,

tax.fossoriality = mapping.fossoriality,

tax.trophic_level = mapping.trophic_level,

tax.foraging_stratum = mapping.foraging_stratum,

tax.activity_cycle = mapping.activity_cycle,

tax.island_endemicity = mapping.island_endemicity

FOREACH (realm in mapping.biogeographical_realm |

MERGE (geo:Geography {name : realm, data_source : "COMBINE" })

MERGE (tax)-[:INHABITS]->(geo)

)

*FluNet:*

UNWIND $Mapping AS mapping

CREATE (flunet:Report {report_id : toInteger(mapping.report_id), data_source: 'FluNet'})

CREATE (event:Event {event_id : mapping.eventId,

data_source : 'FluNet',

start_date : DATE(mapping.start_date),

end_date : DATE(mapping.end_date),

collected : toFloat(mapping.Collected),

processed : toFloat(mapping.Processed)})

MERGE (host:Taxon {tax_id : toInteger(9606),

name : 'Homo sapiens',

rank : 'species',

data_source : 'NCBI Taxonomy'})

MERGE (influenzaA:Taxon {tax_id : toInteger(11320),

name : "Influenza A virus",

rank : "species",

data_source : 'NCBI Taxonomy'})

MERGE (influenzaB:Taxon {tax_id : toInteger(11520),

name : "Influenza B virus",

rank : "species",

data_source : 'NCBI Taxonomy'})

MERGE (influenzaH:Taxon {tax_id : toInteger(114727),

name : "H1N1 subtype",

rank : "serotype",

data_source : 'NCBI Taxonomy'})

MERGE (flunet)-[:REPORTS]->(event)

MERGE (event)-[:INVOLVES {role : 'host'}]->(host)

FOREACH (map in (CASE WHEN mapping.AH1 <> '' THEN [1] ELSE [] END) |

MERGE (event)-[:INVOLVES {role : 'pathogen', subtype : 'A(H1)',

positive : toFloat(mapping.AH1), deaths: 'NA', observation_type : "Laboratory detection"}]->(influenzaA))

FOREACH (map in (CASE WHEN mapping.AH1N1 <> '' THEN [1] ELSE [] END) |

MERGE (event)-[:INVOLVES {role : 'pathogen',

positive : toFloat(mapping.AH1N1), deaths: 'NA', observation_type : "Laboratory detection"}]->(influenzaH))

FOREACH (map in (CASE WHEN mapping.AH3 <> '' THEN [1] ELSE [] END) |

MERGE (event)-[:INVOLVES {role : 'pathogen', subtype : 'A(H3)',

positive : toFloat(mapping.AH3), deaths: 'NA', observation_type : "Laboratory detection"}]->(influenzaA))

FOREACH (map in (CASE WHEN mapping.AH5 <> '' THEN [1] ELSE [] END) |

MERGE (event)-[:INVOLVES {role : 'pathogen', subtype : 'A(H5)',

positive : toFloat(mapping.AH5), deaths: 'NA', observation_type : "Laboratory detection"}]->(influenzaA))

FOREACH (map in (CASE WHEN mapping.Anotsubtyped <> '' THEN [1] ELSE [] END) |

MERGE (event)-[:INVOLVES {role : 'pathogen', subtype : 'NA',

positive : toFloat(mapping.Anotsubtyped), deaths: 'NA', observation_type : "Laboratory detection"}]->(influenzaA))

FOREACH (map in (CASE WHEN mapping.BYamagata <> '' THEN [1] ELSE [] END) |

MERGE (event)-[:INVOLVES {role : 'pathogen', subtype : 'Yamagata',

positive : toFloat(mapping.BYamagata), deaths: 'NA', observation_type : "Laboratory detection"}]->(influenzaB))

FOREACH (map in (CASE WHEN mapping.BVictoria <> '' THEN [1] ELSE [] END) |

MERGE (event)-[:INVOLVES {role : 'pathogen', subtype : 'Victoria',

positive : toFloat(mapping.BVictoria), deaths: 'NA', observation_type : "Laboratory detection"}]->(influenzaB))

FOREACH (map in (CASE WHEN mapping.Bnotsubtyped <> '' THEN [1] ELSE [] END) |

MERGE (event)-[:INVOLVES {role : 'pathogen', subtype : 'NA',

positive : toFloat(mapping.Bnotsubtyped), deaths: 'NA', observation_type : "Laboratory detection"}]->(influenzaB))

FOREACH (map in (CASE WHEN mapping.geonames.geonameId IS NOT NULL THEN [1] ELSE [] END) |

MERGE (territory:Geography {geoname_id : mapping.geonames.geonameId})

ON CREATE SET

territory.data_source = 'GeoNames',

territory.geoname_id = toInteger(mapping.geonames.geonameId),

territory.name = mapping.geonames.name,

territory.admin_type = mapping.geonames.adminType,

territory.iso2 = mapping.geonames.iso2,

territory.fcl_name = mapping.geonames.fclName,

territory.fcode_name = mapping.geonames.fcodeName,

territory.lat = toFloat(mapping.geonames.lat),

territory.long = toFloat(mapping.geonames.lng),

territory.fcode = mapping.geonames.fcode

MERGE (event)-[:OCCURS_IN]->(territory))

*GeoNames:*

UNWIND $Mapping as mapping

MERGE (geo:Geography {geoname_id : toInteger(mapping.geonameId)})

ON CREATE SET

geo.name = mapping.name,

geo.admin_type = mapping.adminType,

geo.iso2 = mapping.iso2,

geo.fcl_name = mapping.fclName,

geo.fcode_name = mapping.fcodeName,

geo.lat = toFloat(mapping.lat),

geo.long = toFloat(mapping.lng),

geo.fcode = mapping.fcode

WITH COLLECT(geo) AS hierarchy

UNWIND RANGE(0, SIZE(hierarchy) - 2) as idx

WITH hierarchy[idx] AS h1, hierarchy[idx+1] AS h2

MERGE (h1)-[:CONTAINS_GEO]->(h2)

*GMPD-2:*

UNWIND $Mapping AS mapping

CREATE (gmpd:Report {data_source: "GMPD",

reference : mapping.Citation,

report_id: toInteger(mapping.report_id)})

CREATE (sample:Sample {data_source: "GMPD", processed: toFloat(mapping.processed)})

MERGE (gmpd)-[:REPORTS]->(sample)

// Process host information

FOREACH (map in (CASE WHEN mapping.Host.taxId IS NOT NULL THEN [1] ELSE [] END) |

MERGE (host:Taxon {tax_id : toInteger(mapping.Host.taxId)})

ON CREATE SET

host.name = mapping.Host.name,

host.rank = mapping.Host.rank,

host.data_source = "NCBI Taxonomy"

MERGE (sample)-[:INVOLVES {role : 'host'}]->(host))

// Process pathogen information

FOREACH (map in (CASE WHEN mapping.Parasite.taxId IS NOT NULL THEN [1] ELSE [] END) |

MERGE (pathogen:Taxon {tax_id : toInteger(mapping.Parasite.taxId)})

ON CREATE SET

pathogen.name = mapping.Parasite.name,

pathogen.rank = mapping.Parasite.rank,

pathogen.data_source = "NCBI Taxonomy"

MERGE (sample)-[:INVOLVES {role : 'pathogen',

observation_type : mapping.SamplingType,

positive : toFloat(mapping.positive),

deaths : "NA",

species_wild : toBoolean(mapping.species_wild)

}]->(pathogen))

// Process geographical location

FOREACH (map in (CASE WHEN mapping.location.geonameId IS NOT NULL THEN [1] ELSE [] END) |

MERGE (territory:Geography {geoname_id : toInteger(mapping.location.geonameId)})

ON CREATE SET

territory.data_source = 'GeoNames',

territory.geoname_id = toInteger(mapping.location.geonameId),

territory.name = mapping.location.name,

territory.admin_type = mapping.location.adminType,

territory.iso2 = mapping.location.iso2,

territory.fcl_name = mapping.location.fclName,

territory.fcode_name = mapping.location.fcodeName,

territory.lat = toFloat(mapping.location.lat),

territory.long = toFloat(mapping.location.lng),

territory.fcode = mapping.location.fcode

MERGE (sample)-[:OCCURS_IN]->(territory))

*NCBI Taxonomy:*

UNWIND $Mapping as mapping

MERGE (tax:Taxon {tax_id : toInteger(mapping.taxId)})

ON CREATE SET

tax.name = mapping.name,

tax.rank = mapping.rank,

tax.data_source = mapping.data_source

WITH COLLECT(tax) AS hierarchy

UNWIND RANGE(0, SIZE(hierarchy) - 2) as idx

WITH hierarchy[idx] AS h1, hierarchy[idx+1] AS h2

MERGE (h1)-[:CONTAINS_TAX]->(h2)

*UN World Pop:*

UNWIND $Mapping AS mapping

CREATE (population:Population {

data_source : mapping.data_source,

report_id : mapping.report_id,

date : DATE(mapping.date),

total_population : toFloat(mapping.TPopulation1July),

median_age : toFloat(mapping.MedianAgePop),

natural_change : toFloat(mapping.NatChange),

population_change : toFloat(mapping.PopChange),

births : toFloat(mapping.Births),

deaths : toFloat(mapping.Deaths),

life_expectancy : toFloat(mapping.LEx),

infant_deaths : toFloat(mapping.InfantDeaths),

under_five_deaths : toFloat(mapping.Under5Deaths),

net_migration : toFloat(mapping.NetMigrations)})

MERGE (geography:Geography {geoname_id: toInteger(mapping.geonames.geonameId)})

MERGE (population)-[:INHABITS]->(geography)

*WAHIS:*

UNWIND $Mapping AS mapping

MERGE (report:Report {report_id: toInteger(mapping.report.reportId)})

ON CREATE SET

report.data_source = "WAHIS",

report.report_date = DATE(mapping.report.reportedOn),

report.reason_for_report = mapping.event.reason.translation,

report.report_description = mapping.event.eventComment

MERGE (event:Event {event_id : mapping.outbreak.outbreakId})

ON CREATE SET

event.start_date = DATE(mapping.outbreak.start_date),

event.end_date = DATE(mapping.outbreak.end_date),

event.description = mapping.outbreak.description,

event.data_source = "WAHIS"

MERGE (report)-[:REPORTS]->(event)

// Set geographical information

MERGE (territory:Geography {geoname_id : toInteger(mapping.outbreak.geonames.geonameId)})

ON CREATE SET

territory.data_source = 'GeoNames',

territory.geoname_id = toInteger(mapping.outbreak.geonames.geonameId),

territory.name = mapping.outbreak.geonames.name,

territory.admin_type = mapping.outbreak.geonames.adminType,

territory.iso2 = mapping.outbreak.geonames.iso2,

territory.fcl_name = mapping.outbreak.geonames.fclName,

territory.fcode_name = mapping.outbreak.geonames.fcodeName,

territory.lat = toFloat(mapping.outbreak.geonames.lat),

territory.long = toFloat(mapping.outbreak.geonames.lng),

territory.fcode = mapping.outbreak.geonames.fcode

MERGE (event)-[:OCCURS_IN]->(territory)

// Set host information (mapping.hosts = array of dictionaries)

// Skip host data list if tax ID is null

FOREACH (hostDataList in mapping.hosts |

FOREACH (hostData in (CASE WHEN hostDataList.taxId IS NOT NULL THEN hostDataList ELSE [] END) |

MERGE (host:Taxon {tax_id: toInteger(hostData.taxId)})

ON CREATE SET

host.name = hostData.name,

host.rank = hostData.rank,

host.data_source = "NCBI Taxonomy"

MERGE (event)-[involves:INVOLVES {role: 'host'}]->(host)

ON CREATE SET

event.processed = toFloat(hostData.processed),

involves.positive = toFloat(hostData.positive),

involves.deaths = toFloat(hostData.deaths),

involves.observation_type = hostData.observation_type,

involves.observation_date = DATE(hostData.observation_date),

involves.species_wild = toBoolean(hostData.species_wild)

))

// Process pathogen information

FOREACH (map in (CASE WHEN mapping.pathogen.taxId IS NOT NULL THEN [1] ELSE [] END) |

MERGE (pathogen:Taxon {tax_id : toInteger(mapping.pathogen.taxId)})

ON CREATE SET

pathogen.name = mapping.pathogen.name,

pathogen.rank = mapping.pathogen.rank,

pathogen.data_source = "NCBI Taxonomy"

MERGE (event)-[:INVOLVES {role : 'pathogen'} ]->(pathogen))

**Supplementary File 3: Queries for analysis**

// HPAI cases by month, country, and mammal species

MATCH (hpai:Taxon)

WHERE hpai.name STARTS WITH "H5" OR hpai.name STARTS WITH "H7"

MATCH (t:Taxon {name:"Mammalia"})-[c:CONTAINS_TAX*]->(mammals:Taxon)

MATCH (hpai)<-[inv:INVOLVES {role: 'pathogen'}]-(event:Event)-[involves:INVOLVES {role:'host'}]->(mammals),

(event)-[o:OCCURS_IN]->(place:Geography)

WHERE DATE(event.start_date) >= DATE("2020-01-01") AND DATE(event.start_date) <= DATE("2023-07-01")

MATCH (place)<-[:CONTAINS_GEO*]-(country:Geography {fcode:"PCLI"})

WITH mammals.name AS mammalName, country.name AS countryName, apoc.temporal.format(event.start_date, 'yyyy-MM') AS month, sum(toInteger(involves.positive)) AS totalCases

WHERE totalCases > 0

RETURN mammalName AS species, countryName AS country, month AS yearMonth, totalCases AS monthlyCases

ORDER BY month ASC

// All influenza A cases by date, place, host species, host class, and pathogen subtype

MATCH (parent:Taxon {name: 'Influenza A virus'})-[:CONTAINS_TAX*0..]->(virus:Taxon)

OPTIONAL MATCH (virus)<-[:INVOLVES {role: 'pathogen'}]-(event:Event)-[involves:INVOLVES {role: 'host'}]->(species:Taxon), (event)-[:OCCURS_IN]->(g:Geography)

OPTIONAL MATCH (species)<-[:CONTAINS_TAX*]-(t:Taxon {rank: "Class"})

WHERE involves.positive IS NOT NULL AND involves.positive <> 'NA' AND species.name IS NOT NULL

WITH g.name AS place, g.lat as lat, g.long as long, event.start_date AS date, SUM(toInteger(involves.positive)) AS totalCases, t.name AS class, species.name as host, parent.name as species, virus.name as subtype

RETURN place, lat, long, date, class, host, totalCases, species, subtype

ORDER BY date DESC
